# DRAF-Net: Quality-Aware RGB–Skeleton Temporal Fusion for Driver Risk-Behavior Recognition

**DOI:** 10.3390/s26206620

**Published:** 2026-10-19

**Authors:** Wei Shen, Xingang Mou, Qun Yuan, Yichao Wang, Peixuan Zhu

**Affiliations:** 1 Key Laboratory of Advanced Manufacturing Technology for High Performance Parts, Ministry of Education, Wuhan 430070, China; wei.shen333@whut.edu.cn (W.S.); sunnymou@whut.edu.cn (X.M.); yuanqun@whut.edu.cn (Q.Y.); wangyichao@whut.edu.cn (Y.W.); 2 School of Mechanical and Electronic Engineering, Wuhan University of Technology, 122 Luoshi Road, Hongshan District, Wuhan 430070, China

**Keywords:** driver behavior recognition, multimodal fusion, RGB–skeleton, quality-aware fusion, bounded correction, early warning

## Abstract

Occlusion in vehicle cabins and pose-estimation errors can reduce the reliability of skeleton information. Conventional fusion methods may propagate unreliable evidence when the RGB, joint, and bone streams produce conflicting predictions. We therefore propose DRAF-Net, a Dual-stage Risk-Aware Fusion Network. The Pose-Quality-guided Risk Bottleneck Temporal Adapter (PQR-BTA) regulates information exchange among the three streams using 12 skeleton-quality measures, prediction confidence, and cross-stream disagreement. Risk Correction Protection (RCP) then uses the baseline prediction as a reference and bounds changes to the high-risk probability and four-level classification scores. Experiments used three mutually exclusive subject splits from Drive&Act, with three random seeds per split and a shared protocol for input features, training, and validation-based model selection. Across the three subject splits, DRAF-Net achieved a high-risk AUPRC of 96.16±2.44%, exceeding the strongest comparison method by 3.13 percentage points. At the validation threshold targeting a FAR of approximately 5%, Recall reached 89.18±5.48%, an improvement of 5.82 points over the strongest comparison method. The deviations are sample standard deviations across the three split-level seed means. These results show that DRAF-Net organizes complementary RGB and skeleton evidence effectively while supporting both the ranking and detection of high-risk samples.

## 1. Introduction

Driver behavior recognition is an important sensing function in intelligent cabins. A practical system must identify both the driver’s current action and actions that may compromise driving safety. RGB video preserves interaction objects such as phones, drinks, and in-vehicle displays, together with the surrounding cabin context. Skeleton sequences describe the motion of the arms, head, and torso. These two sources provide complementary views of driver behavior, but their reliability varies with steering-wheel occlusion, self-occlusion, illumination changes, and keypoint-localization errors. For high-risk behavior, missed detections can delay warnings, whereas false alarms may repeatedly disturb the driver. Overall accuracy alone is therefore insufficient for evaluating practical warning performance [1,2,3].

Multimodal fusion is most useful when it accounts for the conditions that make individual inputs unreliable. In autonomous-vehicle depth estimation, cameras, LiDAR, and radar have complementary failure modes, yet sensor misalignment, computational cost, limited interpretability, domain shift, and real-time constraints remain challenging [4]. Cross-domain visual inspection faces related robustness problems, including occlusion, clutter, scarce annotations, noisy pseudo-labels, and domain shift; recent work identifies multimodal fusion and adaptive strategies as promising directions [5]. Although these tasks differ from in-cabin behavior recognition, both highlight the need to combine RGB and skeleton evidence according to its reliability in each sample.

RGB-based methods use 3D convolution, dual-rate pathways, segment sampling, or video Transformers to extract appearance and motion information. I3D, R(2+1)D, SlowFast, and X3D model action changes with spatiotemporal convolution. TSN captures longer temporal processes through sparse segment sampling, whereas TimeSformer, Video Swin, and MViTv2 extend temporal modeling through attention or multiscale structures [6,7,8,9,10,11,12,13]. These methods directly observe interaction objects and can distinguish actions with similar motion but different manipulated objects. However, when a hand or object is occluded, an RGB model may rely on background or cabin layout, which can reduce generalization across subjects and vehicles.

Skeleton-based methods represent human joints as graphs or heatmaps and focus on body motion. ST-GCN, 2s-AGCN, CTR-GCN, HD-GCN, DGNN, and MS-G3D learn from joint connectivity and temporal variation. PoseC3D, ST-TR, and subsequent methods further strengthen spatiotemporal modeling with heatmaps or Transformers [14,15,16,17,18,19,20,21,22,23]. Skeleton representations are compact and relatively insensitive to color and background changes. However, they do not directly describe phones, cups, or other objects, and they inherit missing keypoints, position jumps, and abnormal bone lengths from pose estimation. Driver-behavior studies have improved recognition through pose descriptors, temporal selection, lightweight networks, and multisource monitoring [24,25,26,27,28]. A skeleton stream alone nevertheless lacks the object evidence required for fine-grained in-cabin actions.

Existing approaches to combining RGB and skeleton evidence can be grouped into three broad categories. The first directly concatenates or weights branch outputs. Such designs are simple but usually apply nearly fixed combinations across samples. The second uses gating, channel modulation, gradient balancing, or modality dropout to adjust each branch dynamically [29,30,31]. The third introduces finer interaction at intermediate feature levels, for example, by transferring scene and pose information frame by frame or aggregating different sources through a small shared representation [32,33]. For multimodal in-cabin video, DFS transfers features across modalities and adjacent frames to strengthen cross-source interaction and temporal propagation. MultiFuser learns relationships among video modalities within local image regions and then aggregates them into a global representation [34,35]. These studies demonstrate the value of dynamic fusion, but most learn weights mainly from features. Few jointly use measurable skeleton quality, predictive confidence, and disagreement among branches.

Most fusion modules can also modify baseline predictions without explicitly controlling the magnitude of the change. When one modality is affected by occlusion, detection error, or noise, the fused result may move too far from the original prediction and increase false alarms or misclassification. DRAF-Net bounds the change between the baseline and fused predictions. This design allows complementary evidence to correct the baseline while limiting the effect of a low-quality modality. The study therefore addresses two related problems: adapting RGB–skeleton information exchange to the reliability of each sample and limiting how far the fused result can move the baseline decision.

We propose DRAF-Net to address these problems. Its PQR-BTA uses 12 skeleton-quality measures computed from OpenPose BODY_25 data. It combines these measures with prediction confidence and cross-stream disagreement to regulate information exchange among the RGB, joint, and bone streams. RCP then uses the baseline prediction as a reference and separately bounds adjustments to the high-risk probability and four-level logits. The two stages connect evidence fusion with controlled decision correction. The remainder of this paper is organized as follows: Section 2 describes DRAF-Net and the four-level risk-recognition task, Section 3 presents the experimental protocol and results, and Section 4 summarizes the main findings.

## 2. Method

We selected 16 of the 34 mid-level actions in Drive&Act [36] that are closely related to driving operations and attention allocation. These actions were assigned to low, medium, high, and conditional risk levels. The assignment primarily considered visual, manual, and cognitive resource demand, together with action duration, interruptibility, and effects on road attention. Actions whose risk could not be determined from the action alone were assessed according to vehicle state, timing, and driving context. These criteria were informed by ISO 17488, the NHTSA visual–manual distraction guidelines, naturalistic driving crash-risk research, and studies of cognitive load [37,38,39,40]. Low risk denotes the absence of an evident secondary task. Medium risk denotes partial resource use by an action that is usually brief or interruptible. High risk denotes sustained visual, manual, or cognitive demand that may impair road monitoring and emergency response. Conditional risk denotes an action whose effect depends on vehicle state and context.

The low-risk class contains *sitting still*. The medium-risk class contains *eating*, *drinking*, *opening/closing bottle*, *preparing food*, *using multimedia display*, and *pressing automation button*. The high-risk class contains *interacting/talking with phone*, *reading magazine/newspaper*, *working on laptop*, and *writing*. The risk of *fastening/unfastening seat belt* depends on whether the vehicle is moving and on the action context, so it is assigned to conditional risk.

We evaluated four-level risk classification and high-risk warning separately. Four-level classification distinguishes low, medium, high, and conditional risk, and the confusion matrix shows the direction of errors between classes. For high-risk warning, high-risk samples are positive and the remaining three classes are negative. Recall is the proportion of high-risk samples detected by the model. The false-alarm rate (FAR) is the proportion of non-high-risk samples that incorrectly trigger a warning. The area under the precision–recall curve (AUPRC) measures high-risk ranking across thresholds. The warning threshold was selected on the validation set to maximize high-risk Recall subject to a FAR of no more than 5%, and was then fixed for test evaluation.

**Figure 1:**
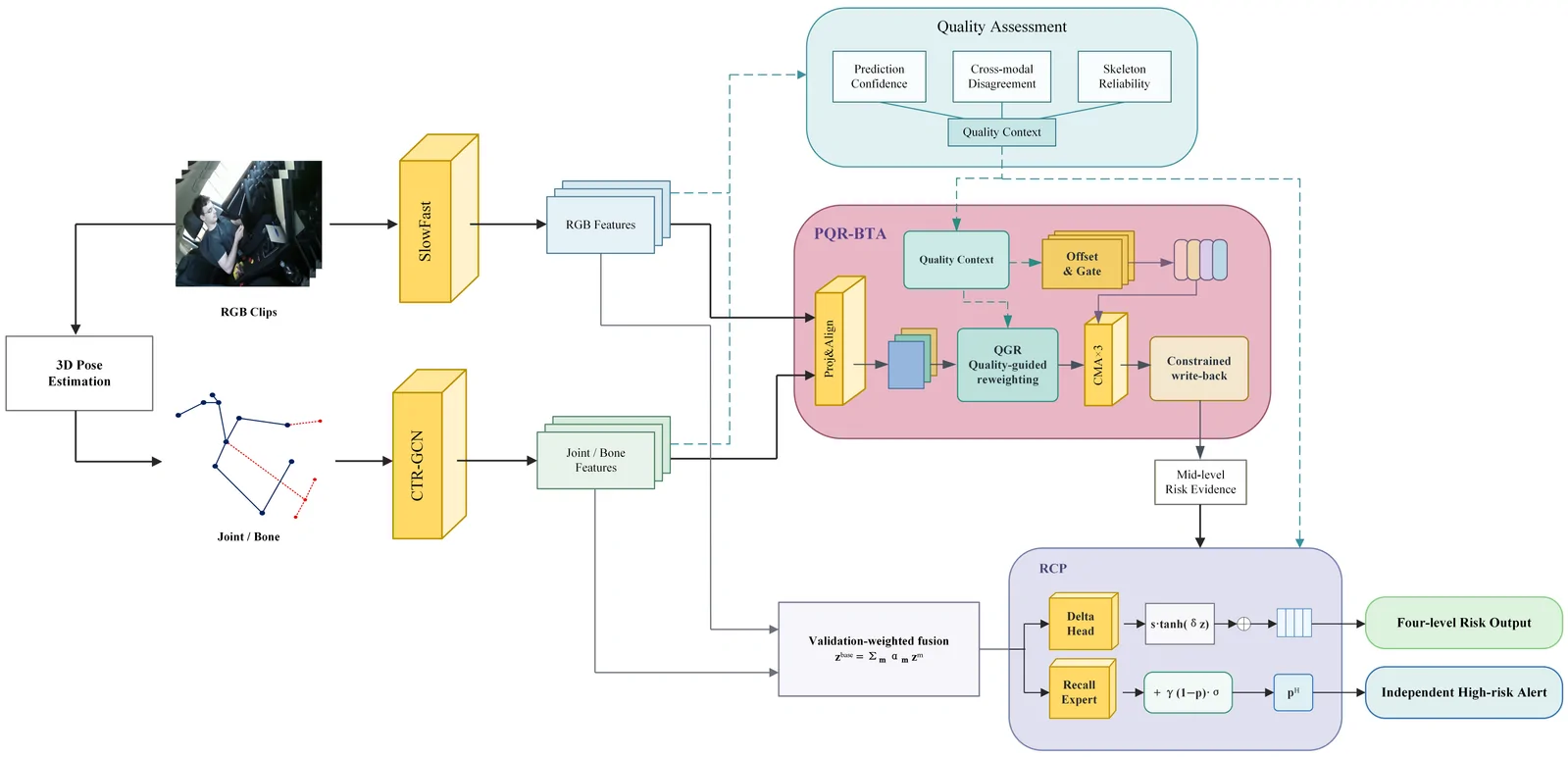
Overall architecture of DRAF-Net. Solid black and gray arrows show the main feature and prediction paths. Dashed cyan and green arrows indicate reliability-context and control paths, while purple arrows indicate the auxiliary-vector path within PQR-BTA. RCP produces four-level risk predictions and an independent high-risk warning.

Driver risk-behavior recognition requires object, hand-action, and local-scene cues from RGB, together with joint motion and action rhythm from skeletons. Fixed-weight fusion cannot respond to changes in skeleton quality or agreement among branches. It may therefore introduce unreliable pose evidence or incidental appearance variation into the final decision. Figure 1 presents the DRAF-Net pipeline. The RGB, joint, and bone streams first produce a baseline risk prediction. PQR-BTA then regulates cross-stream information exchange using skeleton quality, predictive confidence, and branch disagreement. RCP finally produces four-level risk scores and a separate high-risk warning.

Let the feature extractors for the RGB, joint, and bone inputs be $E_{m}:X_{m}\to R^{T_{m}\times d_{m}}$, where m∈{r,j,b}. A position-wise linear projection and temporal alignment operator $P_{m}:R^{T_{m}\times d_{m}}\to R^{16\times 256}$ maps each stream into the shared fusion space. For sample *i*, $H_{i}^{m}$ denotes its temporal feature, ${\bar{h}}_{i}^{m}$ its temporally pooled representation, and $z_{i}^{m}$ its four-level risk logits. A logit is a score before Softmax converts it into a probability. The baseline fusion prediction is

(1) $$H_{i}^{m}=P_{m}\;\left(E_{m}\left(x_{i}^{m}\right)\right),\;z_{i}^{\mathrm{base}}=\sum_{m\in \{r,j,b\}}\alpha_{m}z_{i}^{m},\;\sum_{m}\alpha_{m}=1,\;\alpha_{m}\geq 0.$$

where $\alpha_{m}$ is a modality weight determined only from the validation set. The baseline logits $z_{i}^{\mathrm{base}}$ provide the reference for subsequent correction rather than the final output. This reference prevents the fusion module from making large adjustments that are disconnected from the original modality evidence.

Occlusion, missing hand keypoints, and joint jitter can reduce the stability of skeleton motion cues. The RGB and skeleton streams may also predict different labels for the same action. Motivated by uncertainty-based descriptions of predictive reliability [41,42], PQR-BTA explicitly receives skeleton quality, predictive confidence, and modality disagreement. It uses these signals to construct five learnable auxiliary vectors. Four vectors correspond to the four risk classes, and one supports high-risk warning. They aggregate multimodal evidence related to the current decision rather than introducing another input modality. Evidence exchange uses Transformer self-attention [43,44].

PQR-BTA uses a 12-dimensional skeleton-quality vector $q_{i}\in R^{12}$ to condition cross-modal gating. Skeletons come from the OpenPose 3D data released with Drive&Act and follow the BODY_25 joint order. CTR-GCN encodes the joint stream and a bone stream formed from differences between adjacent joints. Let $x_{itv}\in R^{3}$ be the coordinate of joint *v* at frame *t* in sample *i*, and let $m_{it}\in \left\{0,1\right\}$ be the valid-time mask. A joint is valid when all coordinates are finite and ${∥x_{itv}∥}_{2}>10^{-6}$. The vector contains the following: (1) the zero-coordinate ratio $q_{1}$; (2) the finite-coordinate ratio $q_{2}$; (3) valid-joint ratio $q_{3}$; (4) ratio of frames containing at least one valid joint $q_{4}$; (5) temporal-mask coverage $q_{5}=T^{-1}\sum_{t}m_{it}$; (6) the validity ratio of BODY_25 hand joints 4 and 7, $q_{6}$; (7) the validity ratio of upper-body joints {0,1,2,3,4,5,6,7,15,16,17,18}, $q_{7}$; (8) mean displacement between adjacent valid frames for valid joints, $q_{8}$; (9) standard deviation of this displacement, $q_{9}$; (10) the ratio of displacements exceeding median(d)+3MAD(d), $q_{10}$; (11) the coefficient of variation in all valid BODY_25 bone lengths, $q_{11}=\mathrm{std}\left(L\right)/\max\left(\mathrm{mean}\left(L\right),10^{-6}\right)$; and (12) composite reliability $q_{12}$, defined as

(2) $$\begin{array}{cc} q_{12}={\mathrm{clip}}_{\left[0,1\right]}[ & 0.16\left(1-q_{1}\right)+0.16q_{2}+0.14q_{3}+0.12q_{4}+0.10q_{5}\\ \; & +0.08q_{6}+0.08q_{7}+0.08\left(1-q_{10}\right)+\frac{0.08}{1+q_{11}}]. \end{array}$$

$q_{12}$ is a bounded summary of the selected quality statistics and does not represent an additional sensor signal. The displacement mean $q_{8}$ and standard deviation $q_{9}$ enter the gating network independently. They are excluded from the manual calculation of $q_{12}$ so that uncalibrated displacement scales do not directly alter the bounded reliability score. Within each subject split, all 12 raw statistics are standardized using the training-subset mean $\mu_{q}$ and standard deviation $\sigma_{q}$: ${\tilde{q}}_{i}=\left(q_{i}-\mu_{q}\right)/\left(\sigma_{q}+\epsilon \right)$. The validation and test subsets use the same training-derived parameters. The gating network combines ${\tilde{q}}_{i}$ with the pooled joint and bone features to obtain an RGB channel-modulation vector.

(3) $$g_{i}^{r}=\sigma \;\left(f_{q}\left(\left[{\bar{h}}_{i}^{j};{\bar{h}}_{i}^{b};{\tilde{q}}_{i}\right]\right)\right),\;{\hat{H}}_{i}^{r}=H_{i}^{r}⊙\left[1_{T}{\left(1+\beta_{r}g_{i}^{r}\right)}^{T}\right].$$

Here, $f_{q}\left(\cdot \right)$ is the gating network, σ(·) is the sigmoid function, and $\beta_{r}$ controls the modulation magnitude. Skeleton quality conditions the modulation but does not replace RGB evidence. This design limits the influence of unreliable poses on cross-modal interaction.

After quality-conditioned gating, PQR-BTA concatenates the pooled features, baseline confidence, modality disagreement, and skeleton quality into a context vector $c_{i}$. For any four-level logit vector z, let p=softmax(z) and let $p_{\left(1\right)}$ and $p_{\left(2\right)}$ be the largest and second-largest probabilities. Predictive confidence is defined as

(4) $$\phi \left(z\right)=\left[p_{\left(1\right)},\;1-\frac{-\sum_{k=1}^{4}p_{k}\log\left(p_{k}+10^{-8}\right)}{\log4},\;p_{\left(1\right)}-p_{\left(2\right)}\right].$$

The baseline confidence is $\phi (z_{i}^{\mathrm{base}})$. Let $p_{i}^{r}$, $p_{i}^{j}$, and $p_{i}^{b}$ be the four-level probabilities of the RGB, joint, and bone streams. Their disagreement is

(5) $$d_{i}=\left[\frac{1}{4}{∥p_{i}^{r}-p_{i}^{j}∥}_{1},\;\frac{1}{4}{∥p_{i}^{r}-p_{i}^{b}∥}_{1},\;\frac{1}{4}{∥p_{i}^{j}-p_{i}^{b}∥}_{1}\right].$$

These quantities are computed once for each 3.0-s sample window. Temporal features are mean-pooled using the valid mask, and quality statistics are aggregated within the window. The context $c_{i}=\left[{\bar{h}}_{i}^{r};{\bar{h}}_{i}^{j};{\bar{h}}_{i}^{b};\phi \left(z_{i}^{\mathrm{base}}\right);d_{i};{\tilde{q}}_{i}\right]$ jointly represents multimodal evidence and its reliability. Four class-specific auxiliary vectors and one warning vector are generated from learnable initial representations and bounded offsets. Equation (6) is defined as follows:

(6) $$T_{i}=A+\mathrm{diag}\left(s_{i}\right)\eta \tanh\;\left(F_{\mathrm{off}}\left(c_{i}\right)\right),\;s_{i}=\sigma \;\left(F_{\mathrm{gate}}\left(c_{i}\right)\right).$$

Here, $A\in R^{5\times 256}$ contains the learnable initial representations of the five auxiliary vectors. The multilayer perceptron $F_{\mathrm{off}}:R^{d_{c}}\to R^{5\times 256}$ produces their offsets, and $F_{\mathrm{gate}}:R^{d_{c}}\to R^{5}$ produces five control coefficients. The factor η bounds the offset magnitude. The auxiliary vectors then read temporal evidence from the RGB, joint, and bone streams in sequence and provide bounded feedback to each stream. Equation (7) is defined as follows:

(7) $${\tilde{H}}_{i}^{m}=\mathrm{LN}\;\left({\hat{H}}_{i}^{m}+\rho \left(e\right)s_{\mathrm{inj}}{\bar{g}}_{i}\tanh\left(\Delta H_{i}^{m}\right)\right),\;m\in \left\{r,j,b\right\}.$$

**Figure 2:**
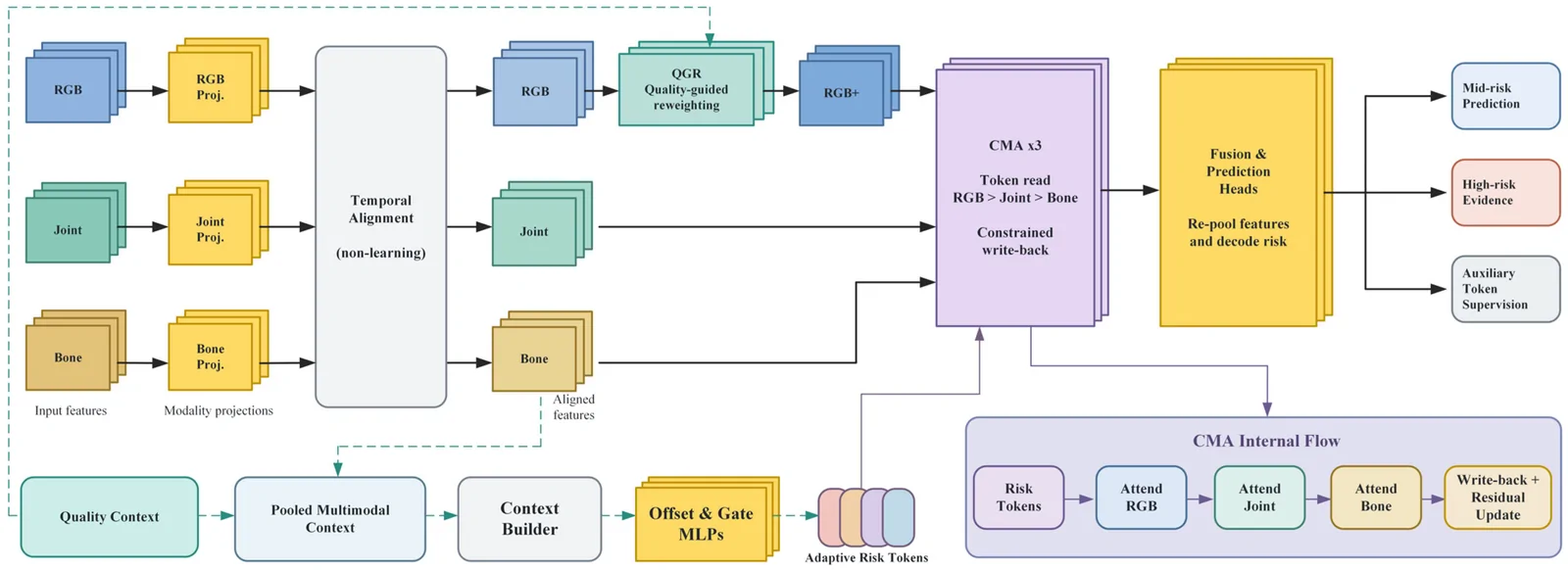
Internal workflow of PQR-BTA. Solid black arrows show feature processing and prediction paths. Dashed teal arrows indicate context construction and quality-conditioned control, while purple arrows show the auxiliary-vector attention path. Blue, green, and yellow feature blocks represent RGB, joint, and bone streams, respectively. The inset illustrates sequential reading of these streams followed by constrained write-back.

Here, $\Delta H_{i}^{m}\in R^{16\times 256}$ is the bounded offset returned to stream *m* after attention-based reading. Layer normalization is $\mathrm{LN}\left(u\right)=\left(u-\mu \right)/\sqrt{\sigma^{2}+\epsilon }⊙g+b$ along the feature dimension. ${\bar{g}}_{i}$ is the mean of the five control coefficients, $s_{\mathrm{inj}}$ is the update scale, and ρ(e) is a warm-up factor that increases with training epoch *e*. The bounded update aggregates complementary evidence while retaining the discriminative information in each stream. Figure 2 shows how the five auxiliary vectors read RGB, joint, and bone evidence and return constrained updates.

Class imbalance or disagreement among modalities can cause ordinary fusion to underestimate high-risk behavior. RCP uses the baseline prediction as a reference and separately corrects high-risk warning and four-level classification. The warning branch preserves an existing high-risk response, whereas the classification branch bounds the change in each class logit.

The independent warning branch preserves the baseline high-risk response while allowing new fusion evidence to increase it. Let $p_{i,\mathrm{base}}^{H}$ be the baseline high-risk probability, and let $\ell_{i,\mathrm{rec}}$ be a supplementary high-risk score estimated from fused features, baseline logits, and confidence features. The high-risk warning probability is

(8) $$p_{i}^{H}=p_{i,\mathrm{base}}^{H}+\gamma \sigma \left(\ell_{i,\mathrm{rec}}\right)\left(1-p_{i,\mathrm{base}}^{H}\right).$$

The cap γ was selected using the validation set from {0.1,0.2,0.3,0.4}. Validation high-risk AUPRC was the primary selection criterion, and Recall at the threshold producing a validation FAR of approximately 5% was used as a secondary criterion when the AUPRC values were close. This search selected γ=0.3, which limits the maximum increment to 30% of the remaining probability space. The test set was not used for parameter selection. Supplementary evidence can therefore strengthen the response to missed high-risk samples while retaining the baseline judgment. This constraint adds positive high-risk evidence without using a negative correction that could suppress an existing high-risk response.

The second RCP branch handles four-level classification without replacing the baseline logits. It uses class-specific bounded residuals so that the permitted adjustment can differ among risk levels. Equation (9) is defined as follows:

(9) $$z_{i}=z_{i}^{\mathrm{base}}+a⊙\tanh\;\left(F_{\mathrm{res}}\left(\left[{\tilde{H}}_{i}^{r};{\tilde{H}}_{i}^{j};{\tilde{H}}_{i}^{b};c_{i}\right]\right)\right).$$

**Figure 3:**
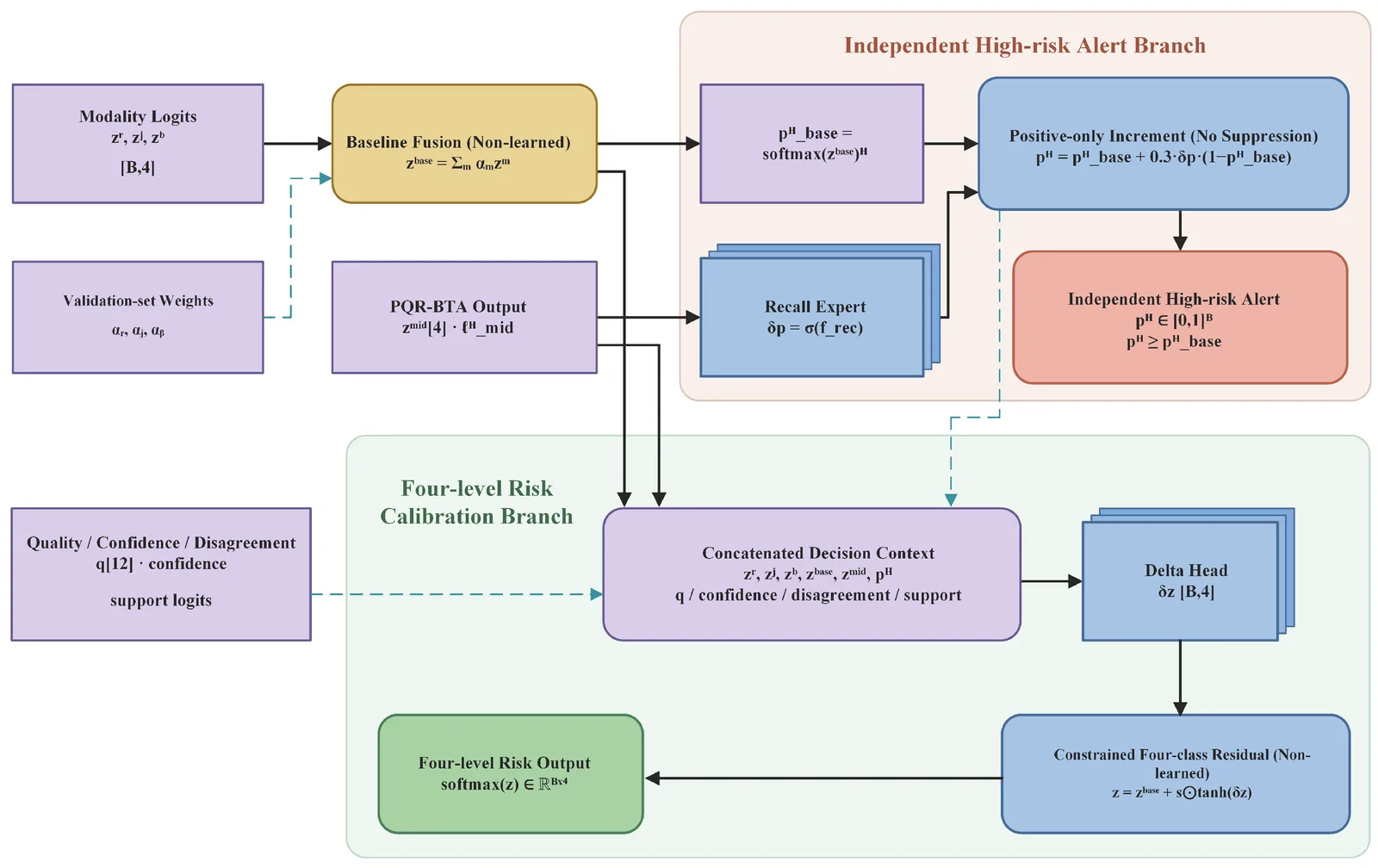
Internal workflow of RCP. Solid black arrows show the main prediction paths. Dashed cyan arrows indicate validation-derived weights and auxiliary decision context. The upper branch produces a bounded, non-negative increment to the baseline high-risk probability. The lower branch applies class-specific bounded residuals to the baseline four-level logits.

Here, $F_{\mathrm{res}}\left(\cdot \right)$ is a multilayer perceptron that maps the concatenated representation to four logit offsets. The relative scale pattern reflects the correction requirements of the four classes. Low and medium risk permit moderate adjustment between adjacent levels, the smaller high-risk scale preserves high-risk evidence already formed by the baseline streams, and conditional risk receives the largest scale because missing vehicle-speed and automation-state signals make this class most context-dependent. Using s=[0.10,0.10,0.03,0.20] as the reference vector, the validation search compared 0.5s, s, and 2s. The same primary and secondary criteria used for γ were applied to select s, giving final scales of 0.10, 0.10, 0.03, and 0.20 for low, medium, high, and conditional risk, respectively. The test set was not used in this search. The tanh(·) operation confines each change to its prescribed range, allowing fusion evidence to correct ambiguous samples without overriding the baseline. Figure 3 summarizes the independent high-risk branch and the class-bounded residual branch.

The training objective jointly constrains four-level classification and high-risk warning. It combines the four-level classification loss, high-risk warning losses, auxiliary supervision, and regularization of the auxiliary vectors. Equation (10) is defined as follows:

(10) $$\begin{array}{cc} L= & L_{\mathrm{risk}}^{\mathrm{CE}}+2L_{\mathrm{high}}^{\mathrm{BCE}}+L_{\mathrm{high}}^{\mathrm{Focal}}+0.1L_{\mathrm{aux}}+0.1L_{\mathrm{vec}}\\ \; & +0.2L_{\mathrm{warn}}+0.01L_{\mathrm{orth}}+0.001L_{\mathrm{off}}. \end{array}$$

$L_{\mathrm{risk}}^{\mathrm{CE}}$ supervises the four risk classes. $L_{\mathrm{high}}^{\mathrm{BCE}}$ and $L_{\mathrm{high}}^{\mathrm{Focal}}$ (Focal loss [45]) emphasize high-risk warning. $L_{\mathrm{aux}}$ and $L_{\mathrm{vec}}$ supervise intermediate representations and auxiliary-vector predictions. $L_{\mathrm{warn}}$ supervises the independent warning vector. $L_{\mathrm{orth}}$ encourages separation among the five auxiliary vectors, and $L_{\mathrm{off}}$ limits their context-dependent offsets. The joint objective retains fine-grained risk classification while maintaining attention to high-risk detection.

For ground-truth label $y_{i}\in \left\{0,1,2,3\right\}$ and high-risk indicator $h_{i}=1\left[y_{i}=2\right]$, the weighted four-class cross-entropy is $L_{\mathrm{risk}}^{\mathrm{CE}}=-N^{-1}\sum_{i}w_{y_{i}}\log\mathrm{softmax}{\left(z_{i}\right)}_{y_{i}}$. Let $l_{i}=\mathrm{logit}\left(p_{i}^{H}\right)=\log\left[p_{i}^{H}/\left(1-p_{i}^{H}\right)\right]$ be the logit of the independent high-risk probability. Binary cross-entropy is computed as $\mathrm{BCEWithLogits}(l_{i},h_{i})$, with a positive-class weight estimated from the training subset. The Focal term uses the same $l_{i}$ and is $-N^{-1}\sum_{i}{\left(1-p_{t,i}\right)}^{2}\log p_{t,i}$, where $p_{t,i}=\sigma \left(l_{i}\right)$ for positive samples and $1-\sigma (l_{i})$ for negative samples. No additional positive-class weight is applied to the Focal term. The auxiliary loss averages intermediate four-class cross-entropy and intermediate high-risk binary cross-entropy. The vector loss is weighted cross-entropy over the four class-specific vectors, and the warning loss is weighted binary cross-entropy for the warning vector. The orthogonality loss penalizes correlations among normalized auxiliary vectors, whereas the offset loss penalizes excessive deviation from their initial representations. Equation (10) gives all active weights; the unused consistency term has zero weight. All class weights are estimated from the current training subset.

## 3. Experiments and Results

The inputs comprised SlowFast RGB features, CTR-GCN joint and bone features, and a 12-dimensional skeleton-quality vector for each 3.0-s window. The three subject-independent test splits contained 1772, 1222, and 1683 samples, giving 4677 samples in total. Each split was evaluated with three random seeds. Table 1 reports the unweighted mean and sample SD across the three split-level seed means. Table 2 reports the mean ± sample SD across seeds within each split. Thus, the first table summarizes variation across subject splits, whereas the second isolates variation from optimization and initialization within a fixed split.

The models were implemented in PyTorch 2.5.1+cu121. The feature extractors were frozen, and only the fusion modules were trained. All methods used AdamW, weighted cross-entropy, a maximum of 30 epochs, batch size 32, early stopping with a patience of 8, and checkpoint selection based on validation high-risk AUPRC. The four comparison heads used a learning rate of $10^{-3}$, a weight decay of $10^{-4}$, a hidden dimension of 256, and a dropout of 0.2. DRAF-Net used a learning rate of $10^{-4}$, the same weight decay and hidden dimension, and a dropout of 0.3. Its objective additionally included high-risk binary cross-entropy, Focal loss, auxiliary classification, and structural regularization. PQR-BTA used four attention heads: internal dropout, auxiliary-vector dropout, and stochastic depth were each 0.1, and the information-update scale was 0.05.

The validation search considered γ∈{0.1,0.2,0.3,0.4}, residual-scale multipliers {0.5,1,2}, and target FARs {1%,3%,5%,10%,15%}. We selected γ and the residual scales primarily by validation high-risk AUPRC, using Recall at approximately 5% validation FAR when AUPRC values were close. Validation data were used for model selection, calibration, and threshold selection; test data were reserved for final evaluation. Across methods, subject splits, input features, labels, random seeds, training budget, early-stopping rule, checkpoint criterion, and evaluation procedure were identical. The methods differed in fusion architecture, associated losses, learning rate, and dropout, as specified above.

Table 1 compares four trained fusion heads: a single-head MLP, a temporal convolutional head, GMU, and attention fusion. DRAF-Net achieved a mean AUPRC of 96.16%, exceeding the TCN risk head by 3.13 points. Its mean Recall@ValFAR5 was 89.18%, 5.82 points above attention fusion. The mean realized test FAR was 5.75%, with variation across subject splits reported in the table.

Under matched splits and seeds, DRAF-Net’s mean AUPRC exceeded that of attention fusion by 3.70 points. Variation across seeds was small within each split, while differences between subject splits were larger, particularly for test FAR.

**Figure 4:**
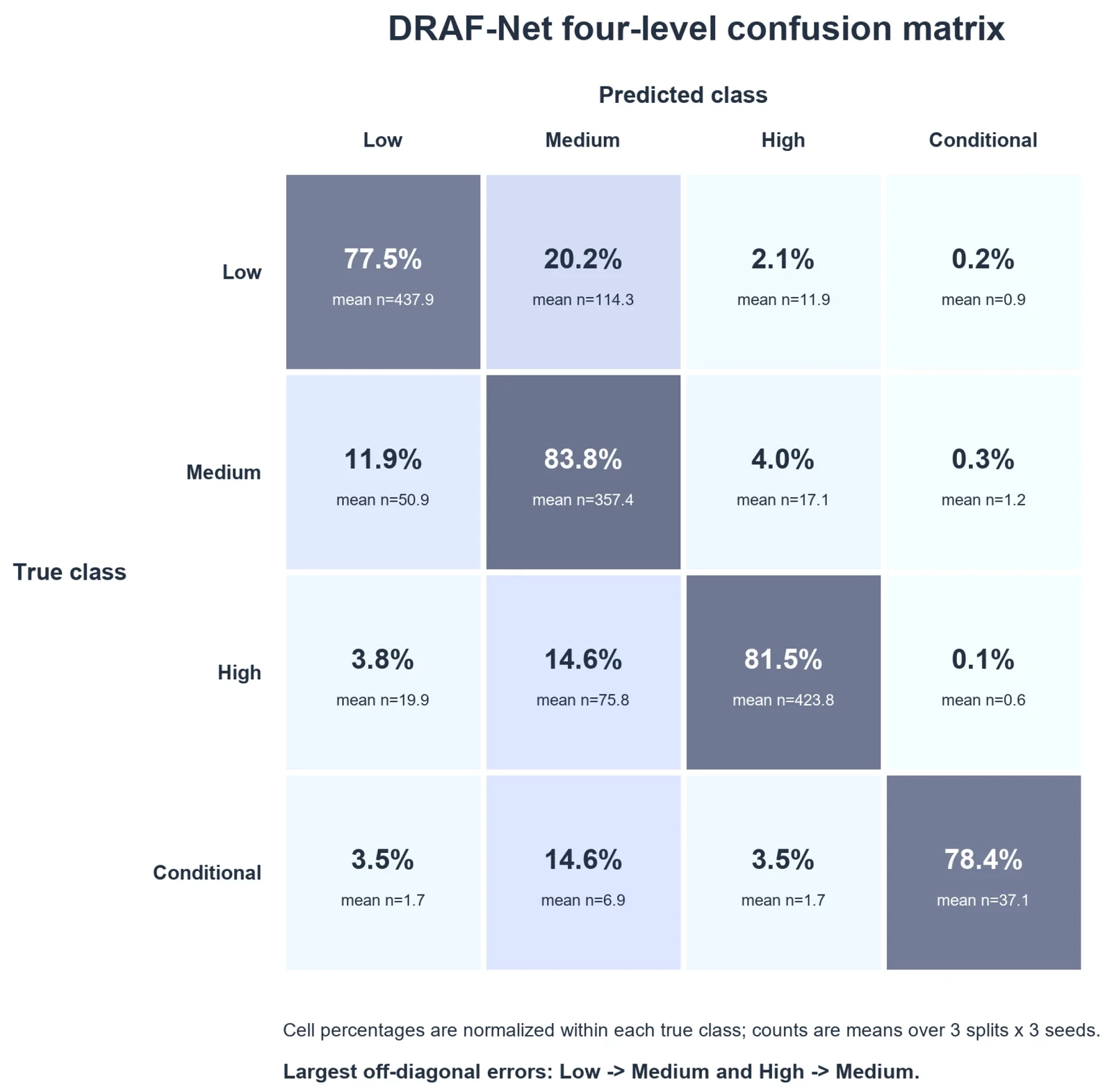
Four-level risk confusion matrix of DRAF-Net.

Figure 4 summarizes the four-level risk-classification errors, with the largest off-diagonal confusions occurring from low to medium risk and from high to medium risk.

The “Base only” configuration uses a validation-selected weighted average of the frozen RGB, joint, and bone logits, without a trainable fusion head. Its mean AUPRC (95.71%) exceeded those of all four trained heads in Table 1. This result indicates that the frozen branches already provide complementary predictions that a simple weighted combination can exploit. The trained heads introduce additional degrees of freedom, which may capture split-specific patterns or disrupt useful branch evidence under the current training setup. We therefore treat PQR-BTA and RCP as incremental refinements to a strong baseline, rather than evidence that learned fusion is inherently superior.

Table 3 compares Base-only, PQR-only, RCP-only, and full DRAF-Net. Relative to Base-only, the mean AUPRC/Recall changes were +0.31/+0.70 points for PQR-only, +0.45/+0.57 for RCP-only, and +0.45/+0.62 for the full model. The full model matched RCP-only in AUPRC and trailed PQR-only in Recall by 0.08 points. These small, metric-dependent differences do not indicate synergy, but they confirm that the full configuration retained a modest gain over Base-only.

Table 4 and Table 5 summarize all tested RCP settings. Both γ and the residual scale were selected using validation data; the corresponding test results are reported only to assess post-selection robustness. Across γ values, mean test AUPRC varied by 0.37 points and Recall@ValFAR5 by 1.32 points. Across residual-scale multipliers, the corresponding ranges were 0.06 and 0.76 points. Table 5 shows the expected operating-point trade-off: increasing the target validation FAR increased both test Recall and realized test FAR.

**Figure 5:**
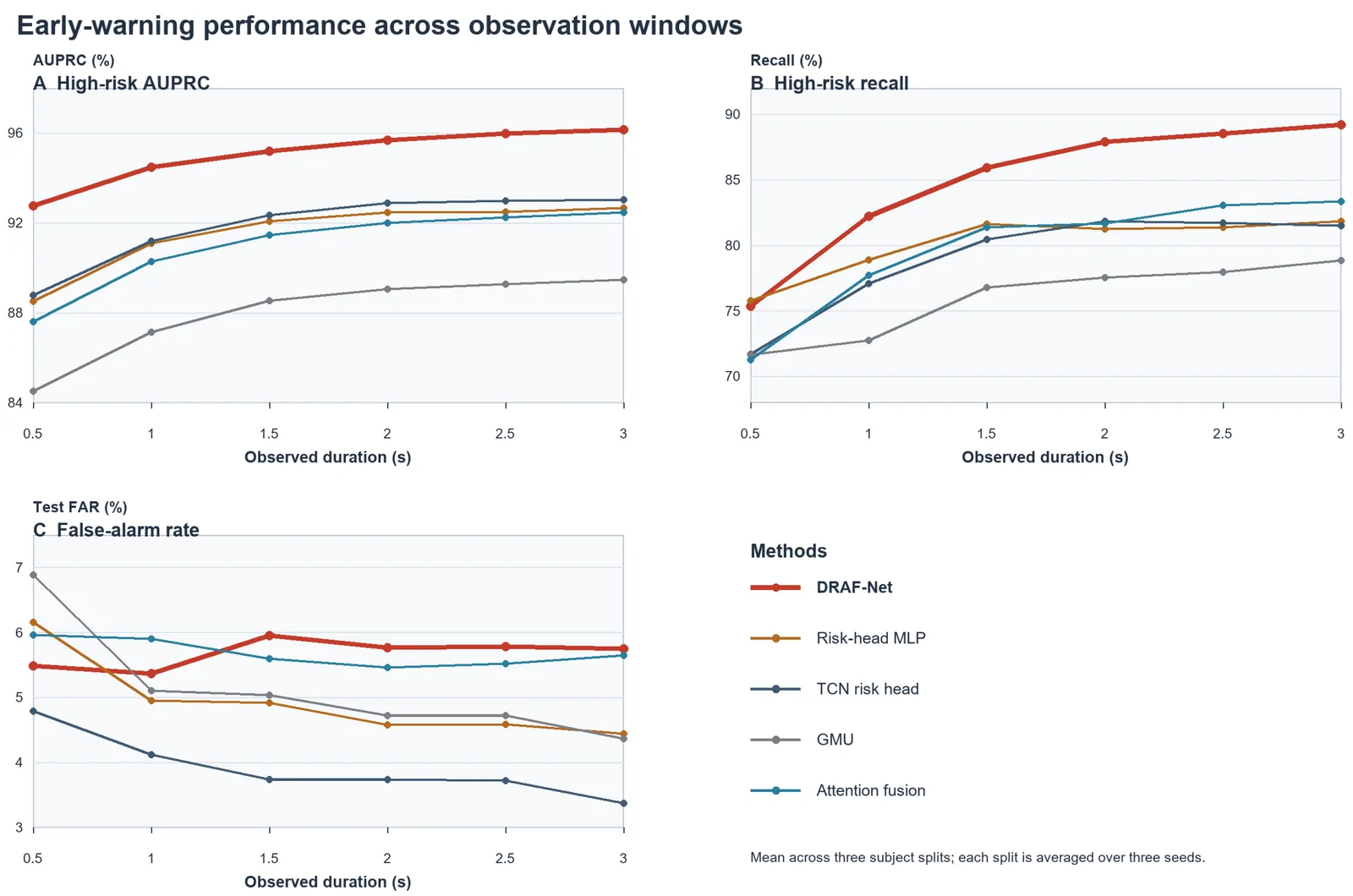
Early-warning results for the compared methods.

The early-warning experiment assessed high-risk recognition before the action was fully observed. It used six observation windows of 0.5, 1.0, 1.5, 2.0, 2.5, and 3.0 s. For the first five windows, the original video frames and pose CSV files were cropped at the current time. Interpolation, resampling, and feature extraction used only data available up to that point. The 3.0-s window used the cache from the main experiment. Shorter-window features were causally re-extracted in a supplemental environment. Full-window checks of that environment did not achieve strict numerical parity with the main feature cache, so the comparison includes an environment boundary at the 3.0-s endpoint. Consequently, the RGB, skeleton, and 12-dimensional quality features contained no future frames. DRAF-Net and the four comparison methods were recomputed across three subject splits and three random seeds using the same validation-threshold rule. Figure 5 shows that AUPRC and Recall generally increased as observation time extended from 0.5 to 3.0 s, whereas the test FAR changed only slightly. These results indicate that DRAF-Net retained useful high-risk ranking and detection capability when only partial action information was available.

## 4. Conclusions

DRAF-Net combines reliability-aware feature exchange with bounded prediction correction for driver risk-behavior recognition. PQR-BTA adjusts interactions among RGB, joint, and bone features using skeleton quality, predictive confidence, and branch disagreement. RCP constrains how far the fused prediction can move from the baseline. Under the matched protocol, DRAF-Net achieved the highest mean AUPRC and Recall among the evaluated fusion heads. Base-only remained competitive, and the stage-level ablation did not indicate synergy: RCP-only matched the full model in AUPRC, whereas PQR-only gave slightly higher Recall. Accordingly, the full model provides a modest improvement over a strong baseline by combining reliability-aware exchange with bounded correction. The early-warning results also show that the model can make useful predictions from partial observations before an action is complete. 

## Figures and Tables

**Table 1:** Overall performance under the matched protocol. Values are the mean ± sample SD across the three split-level seed means (%).

Method	AUPRC	Recall@ValFAR5	Test FAR
Risk-head MLP	92.66±4.80	81.84±3.35	4.44±3.74
TCN risk head	93.03±5.45	81.52±6.88	3.37±2.67
GMU	89.47±6.02	78.84±6.02	4.36±2.15
Attention fusion	92.46±4.49	83.37±4.80	5.64±4.32
**DRAF-Net**	96.16±2.44	89.18±5.48	5.75±6.31

Bold method names identify the proposed method.

**Table 2:** Performance within each subject split over three random seeds (mean ± sample SD, %).

Method	AUPRC			Recall@ValFAR5		
	**Split 0**	**Split 1**	**Split 2**	**Split 0**	**Split 1**	**Split 2**
Risk-head MLP	87.97±0.66	97.56±0.22	92.47±0.41	81.60±2.15	85.30±0.45	78.62±3.99
TCN risk head	87.84±2.91	98.71±0.24	92.54±1.08	78.52±2.31	89.39±2.45	76.64±0.45
GMU	84.07±3.08	95.96±0.55	88.39±2.25	73.83±4.83	85.52±4.27	77.18±4.11
Attention fusion	88.01±1.96	96.99±0.80	92.37±1.26	85.74±3.08	86.52±2.57	77.84±3.78
**DRAF-Net**	95.13±0.06	98.94±0.10	94.40±0.30	95.12±0.39	88.10±1.02	84.32±0.94

Bold method names identify the proposed method.

**Table 3:** Component ablation under the matched protocol. Values are the mean ± sample SD across the three split-level seed means (%).

Configuration	AUPRC	Recall@ValFAR5	Test FAR
Base-only	95.71±2.47	88.56±6.10	6.43±6.49
PQR-only	96.03±2.52	89.26±3.66	5.40±5.20
RCP-only	96.16±2.49	89.13±4.29	4.91±4.86
**Full DRAF-Net**	96.16±2.44	89.18±5.48	5.75±6.31

Bold method names identify the proposed method.

**Table 4:** Post-selection sensitivity of test performance to the RCP cap and residual-scale multiplier (%).

Parameter	Setting	AUPRC	Recall@ValFAR5	Test FAR
γ	0.1	95.89	88.33	4.84
γ	0.2	96.03	89.05	5.72
γ	0.3 (selected)	96.16	89.18	5.75
γ	0.4	96.25	89.66	5.67
Residual scales	0.5s	96.12	89.79	6.03
Residual scales	s (selected)	96.16	89.18	5.75
Residual scales	2s	96.09	89.03	5.46

**Table 5:** DRAF-Net sensitivity to the warning operating point selected on the validation set (%).

Target Validation FAR	Realized Validation FAR	Test Recall	Test FAR
1	0.79	73.33	1.53
3	2.82	85.02	4.23
5 (selected)	4.58	89.18	5.75
10	8.86	94.03	10.10
15	13.09	96.11	14.03

## Data Availability

The publicly available Drive&Act dataset analyzed in this study can be accessed through the official project website at https://driveandact.com/ (accessed on 29 August 2026).

## References

[1] Gao F., Ge X., Li J., Fan Y., Li Y., Zhao R. (2024). Intelligent Cockpits for Connected Vehicles: Taxonomy, Architecture, Interaction Technologies, and Future Directions. Sensors.

[2] Yan X., He J., Wu G., Zhang C., Wang C. (2022). A Proactive Recognition System for Detecting Commercial Vehicle Driver’s Distracted Behavior. Sensors.

[3] Kim W., Jung W.S., Choi H.K. (2019). Lightweight Driver Monitoring System Based on Multi-Task MobileNets. Sensors.

[4] Umar M., Shafi I., Ahmed N., ur Rehman H.M.R., Ashraf I. (2026). Advances in Multi-Sensor Fusion for Depth Estimation in Autonomous Vehicles: A Comprehensive Survey. Adv. Eng. Inform..

[5] Khan S.M., Shafi I., Mehmood M.Z., Caglar A., Naseem M.T., ur Rehman H.M.R., Ashraf I. (2026). Advances in Semi and Self-Supervised Learning Techniques for Cross-Domain Visual Inspection Tools. Int. J. Mach. Learn. Cybern..

[6] Feichtenhofer C., Fan H., Malik J., He K. SlowFast Networks for Video Recognition. Proceedings of the IEEE/CVF International Conference on Computer Vision.

[7] Feichtenhofer C. X3D: Expanding Architectures for Efficient Video Recognition. Proceedings of the IEEE/CVF Conference on Computer Vision and Pattern Recognition.

[8] Li Y., Wu C.Y., Fan H., Mangalam K., Xiong B., Malik J., Feichtenhofer C. MViTv2: Improved Multiscale Vision Transformers for Classification and Detection. Proceedings of the IEEE/CVF Conference on Computer Vision and Pattern Recognition.

[9] Carreira J., Zisserman A. Quo Vadis, Action Recognition? A New Model and the Kinetics Dataset. Proceedings of the IEEE Conference on Computer Vision and Pattern Recognition.

[10] Wang L., Xiong Y., Wang Z., Qiao Y., Lin D., Tang X., Van Gool L. Temporal Segment Networks: Towards Good Practices for Deep Action Recognition. Proceedings of the European Conference on Computer Vision.

[11] Tran D., Wang H., Torresani L., Ray J., LeCun Y., Paluri M. A Closer Look at Spatiotemporal Convolutions for Action Recognition. Proceedings of the IEEE Conference on Computer Vision and Pattern Recognition.

[12] Bertasius G., Wang H., Torresani L. Is Space-Time Attention All You Need for Video Understanding?. Proceedings of the 38th International Conference on Machine Learning.

[13] Liu Z., Ning J., Cao Y., Wei Y., Zhang Z., Lin S., Hu H. Video Swin Transformer. Proceedings of the IEEE/CVF Conference on Computer Vision and Pattern Recognition.

[14] Yan S., Xiong Y., Lin D. Spatial Temporal Graph Convolutional Networks for Skeleton-Based Action Recognition. Proceedings of the Thirty-Second AAAI Conference on Artificial Intelligence.

[15] Shi L., Zhang Y., Cheng J., Lu H. Two-Stream Adaptive Graph Convolutional Networks for Skeleton-Based Action Recognition. Proceedings of the IEEE/CVF Conference on Computer Vision and Pattern Recognition.

[16] Chen Y., Zhang Z., Yuan C., Li B., Deng Y., Hu W. Channel-Wise Topology Refinement Graph Convolution for Skeleton-Based Action Recognition. Proceedings of the IEEE/CVF International Conference on Computer Vision.

[17] Lee J., Lee M., Lee D., Lee S. Hierarchically Decomposed Graph Convolutional Networks for Skeleton-Based Action Recognition. Proceedings of the IEEE/CVF International Conference on Computer Vision.

[18] Shi L., Zhang Y., Cheng J., Lu H. Skeleton-Based Action Recognition with Directed Graph Neural Networks. Proceedings of the IEEE/CVF Conference on Computer Vision and Pattern Recognition.

[19] Liu Z., Zhang H., Chen Z., Wang Z., Ouyang W. Disentangling and Unifying Graph Convolutions for Skeleton-Based Action Recognition. Proceedings of the IEEE/CVF Conference on Computer Vision and Pattern Recognition.

[20] Duan H., Zhao Y., Chen K., Lin D., Dai B. Revisiting Skeleton-Based Action Recognition. Proceedings of the IEEE/CVF Conference on Computer Vision and Pattern Recognition.

[21] Plizzari C., Cannici M., Matteucci M. (2021). Skeleton-Based Action Recognition via Spatial and Temporal Transformer Networks. Comput. Vis. Image Underst..

[22] Song Y.F., Zhang Z., Shan C., Wang L. (2023). Constructing Stronger and Faster Baselines for Skeleton-Based Action Recognition. IEEE Trans. Pattern Anal. Mach. Intell..

[23] Huo R., Chen J., Zhang Y., Gao Q. (2025). 3D Skeleton Aware Driver Behavior Recognition Framework for Autonomous Driving System. Neurocomputing.

[24] Jiao S.J., Liu L.Y., Liu Q. (2021). A Hybrid Deep Learning Model for Recognizing Actions of Distracted Drivers. Sensors.

[25] Abouelnaga Y., Eraqi H.M., Moustafa M.N. (2017). Real-Time Distracted Driver Posture Classification. arXiv.

[26] Eraqi H.M., Abouelnaga Y., Saad M.H., Moustafa M.N. (2019). Driver Distraction Identification with an Ensemble of Convolutional Neural Networks. J. Adv. Transp..

[27] Kashevnik A., Lashkov I., Gurtov A. (2020). Methodology and Mobile Application for Driver Behavior Analysis and Accident Prevention. IEEE Trans. Intell. Transp. Syst..

[28] Ortega J.D., Kose N., Cañas P., Chao M.-A., Unnervik A., Nieto M., Otaegui O., Salgado L. DMD: A Large-Scale Multi-Modal Driver Monitoring Dataset for Attention and Alertness Analysis. Proceedings of the European Conference on Computer Vision Workshops.

[29] Baltrušaitis T., Ahuja C., Morency L.P. (2019). Multimodal Machine Learning: A Survey and Taxonomy. IEEE Trans. Pattern Anal. Mach. Intell..

[30] Joze H.R.V., Shaban A., Iuzzolino M.L., Koishida K. MMTM: Multimodal Transfer Module for CNN Fusion. Proceedings of the IEEE/CVF Conference on Computer Vision and Pattern Recognition.

[31] Wang W., Tran D., Feiszli M. What Makes Training Multi-Modal Classification Networks Hard?. Proceedings of the IEEE/CVF Conference on Computer Vision and Pattern Recognition.

[32] Li J., Sun J., Li Z., Zhang J., Zhuo L. (2026). Multimodal Driver Behavior Recognition Based on Frame-Adaptive Convolution and Feature Fusion. Comput. Vis. Image Underst..

[33] Nagrani A., Yang S., Arnab A., Jansen A., Schmid C., Sun C. Attention Bottlenecks for Multimodal Fusion. Proceedings of the Advances in Neural Information Processing Systems.

[34] Lin D., Lee P.H.Y., Li Y., Wang R., Yap K.H., Li B., Ngim Y.S. Multi-Modality Action Recognition Based on Dual Feature Shift in Vehicle Cabin Monitoring. Proceedings of the ICASSP 2024–2024 IEEE International Conference on Acoustics, Speech and Signal Processing.

[35] Wang R., Wang W., Gao J., Lin D., Yap K.H., Li B. MultiFuser: Multimodal Fusion Transformer for Enhanced Driver Action Recognition. Proceedings of the 2024 IEEE 26th International Workshop on Multimedia Signal Processing.

[36] Martin M., Roitberg A., Haurilet M., Horne M., Reiß S., Voit M., Stiefelhagen R. Drive&Act: A Multi-Modal Dataset for Fine-Grained Driver Behavior Recognition in Autonomous Vehicles. Proceedings of the IEEE/CVF International Conference on Computer Vision.

[37] National Highway Traffic Safety Administration (2013). Visual-Manual NHTSA Driver Distraction Guidelines for In-Vehicle Electronic Devices. Fed. Regist..

[38] (2016). Road Vehicles—Transport Information and Control Systems—Detection-Response Task (DRT) for Assessing Attentional Effects of Cognitive Load in Driving.

[39] Dingus T.A., Guo F., Lee S., Antin J.F., Perez M., Buchanan-King M., Hankey J. (2016). Driver Crash Risk Factors and Prevalence Evaluation Using Naturalistic Driving Data. Proc. Natl. Acad. Sci. USA.

[40] Strayer D.L., Johnston W.A. (2001). Driven to Distraction: Dual-Task Studies of Simulated Driving and Conversing on a Cellular Telephone. Psychol. Sci..

[41] Gal Y., Ghahramani Z. Dropout as a Bayesian Approximation: Representing Model Uncertainty in Deep Learning. Proceedings of the 33rd International Conference on Machine Learning.

[42] Kendall A., Gal Y. What Uncertainties Do We Need in Bayesian Deep Learning for Computer Vision?. Proceedings of the Advances in Neural Information Processing Systems.

[43] Vaswani A., Shazeer N., Parmar N., Uszkoreit J., Jones L., Gomez A.N., Kaiser Ł., Polosukhin I. Attention Is All You Need. Proceedings of the Advances in Neural Information Processing Systems.

[44] Dosovitskiy A., Beyer L., Kolesnikov A., Weissenborn D., Zhai X., Unterthiner T., Dehghani M., Minderer M., Heigold G., Gelly S. An Image Is Worth 16 × 16 Words: Transformers for Image Recognition at Scale. Proceedings of the International Conference on Learning Representations.

[45] Lin T.Y., Goyal P., Girshick R., He K., Dollár P. Focal Loss for Dense Object Detection. Proceedings of the IEEE International Conference on Computer Vision.

